# Longitudinal assessment of radiosurgery response in small brain metastases: AI‐driven precision tumor segmentation and monitoring on serial MRI

**DOI:** 10.1002/mp.70273

**Published:** 2026-01-13

**Authors:** Nauman Bashir Bhatti, James Stewart, Brige Chugh, Jay Detsky, Chia‐Lin Tseng, Chris Heyn, Pejman J. Maralani, Arjun Sahgal, Hany Soliman, Ali Sadeghi‐Naini

**Affiliations:** ^1^ Department of Electrical Engineering and Computer Science Lassonde School of Engineering York University Toronto ON Canada; ^2^ Physical Sciences Platform Sunnybrook Research Institute Sunnybrook Health Sciences Centre Toronto ON Canada; ^3^ Department of Radiation Oncology Odette Cancer Centre Sunnybrook Health Sciences Centre Toronto ON Canada; ^4^ Department of Medical Physics Odette Cancer Centre Sunnybrook Health Sciences Centre Toronto ON Canada; ^5^ Department of Radiation Oncology Temerty Faculty of Medicine University of Toronto Toronto ON Canada; ^6^ Department of Medical Imaging Sunnybrook Health Sciences Centre Toronto ON Canada; ^7^ Department of Medical Imaging, Temerty Faculty of Medicine University of Toronto Toronto ON Canada

**Keywords:** brain metastasis, deep learning, longitudinal tumor segmentation, neighborhood attention mechanism, radiotherapy outcome assessment, serial MRI, transformers

## Abstract

**Background:**

The conventional method of assessing radiotherapy outcome in brain metastases (BM) is based on monitoring tumor size alterations on serial magnetic resonance imaging (MRI). To accurately determine changes in tumor dimensions, targets require delineations on several volumetric images acquired before treatment and at multiple follow‐up scans after radiotherapy. However, manual tumor delineation on serial MRI is labor‐intensive, imposes a significant burden on the clinical workflow, and is prone to variability especially for smaller lesions.

**Purpose:**

This study proposes a novel multi‐step transformer‐based automated framework with a 3D neighborhood attention mechanism, specifically designed to enhance the segmentation precision for BM of various sizes on standard longitudinal MRI. This framework leverages the hierarchical encoding capabilities of transformer architecture to capture intricate tumor characteristics, with a particular focus on improving the delineation of small metastases (<1 cm), which are often overlooked by existing models.

**Methods:**

The proposed framework was trained on the BraTS and BraTS‐METS datasets and evaluated on independent external data acquired from 212 patients (508 BM lesions) treated with stereotactic radiosurgery. The framework's performance was evaluated in segmenting tumors across various size categories, monitoring post‐treatment changes in tumor size on serial MRI, and automatically detecting local control/failure (LC/LF) and adverse radiation effect (ARE) following radiosurgery.

**Results:**

The framework achieved a dice score of 89.8 ± 3.4%, 92.0 ± 3.0%, and 93.1 ± 2.3% for tumors with a size of less than 1 cm, between 1 and 2 cm, and larger than 2 cm, respectively. It also demonstrated high performance in longitudinal monitoring of tumor size changes and in detecting LC/LF and ARE, achieving accuracies greater than 96% across different tumor size categories compared to the clinical outcome assessment. The results exhibited a substantial improvement over state‐of‐the‐art segmentation models, particularly for smaller lesions.

**Conclusions:**

This study represents a step forward toward deploying AI‐driven decision support tools to the neuro‐oncology workflow, reducing the assessment burden on oncologists, and improving consistency in routine radiotherapy outcome assessments.

## 1 INTRODUCTION

Brain metastases (BM) are secondary tumors that originate from primary cancers in other organs before spreading to the brain. They occur in approximately 20%–40% of all cancer patients, with a notably higher incidence in lung, breast, and skin cancer cases.^1^ Patients diagnosed with brain metastasis suffer from poor prognoses. The median survival for patients undergoing treatment ranges between 3 months and 4 years based on the subgroup and origin of the cancer.^2^

The available therapeutic options for treating BM include surgical resection, radiotherapy, chemotherapy, targeted therapy, and immunotherapy.^3^ The options for radiotherapy include whole‐brain radiation therapy (WBRT),^4^ and single‐fraction or hypo‐fractionated stereotactic radiosurgery (SRS).^5^, ^6^ Due to the adverse side effects associated with WBRT, there has been a shift toward using SRS over the past decades, particularly for patients diagnosed with less than 10 brain metastases.^7^ In SRS, a high dose of radiation is precisely delivered to the targeted area over a single session (single‐fraction SRS) or very few sessions (hypo‐fractionated SRS) typically spanning a few days to a week.

Magnetic resonance imaging (MRI) is the primary imaging modality used for diagnosing, treatment planning, and assessing therapy outcomes in BM.^8^ The contrast‐enhanced T1‐weighted (T1c) and T2‐weighted fluid‐attenuated inversion recovery (T2‐FLAIR) images are routinely acquired before radiotherapy (baseline) for treatment planning. This procedure also involves precise delineation of the tumor, typically carried out by experienced radiation oncologists and neuroradiologists to ensure accurate treatment targeting. The T1c and T2‐FLAIR images are also acquired at multiple follow‐up sessions after radiation therapy for outcome assessment. The evaluation of radiotherapy outcomes in brain metastasis is primarily based on standardized criteria established by the response assessment in neuro‐oncology brain metastases (RANO‐BM) working group.^9^ These criteria focus on measuring the longest diameter of the tumor in the axial, coronal, and sagittal planes of MRI. The tumor response is categorized into four main groups: complete response (CR), where no measurable tumor remains; partial response (PR), characterized by a reduction of over 30% in the longest diameter compared to baseline; stable disease (SD), where the tumor does not demonstrate a reduction of over 30% compared to baseline nor does it show an increase of over 20% compared to nadir; and progressive disease (PD), which is defined by an increase of more than 20% in the longest diameter compared to nadir. Clinically, the CR, PR, and SD indicate a local control (LC) outcome, while the PD specifies local failure (LF).

A common radiographic finding associated with SRS is adverse radiation effect (ARE).^10^ This complication arises from high‐dose radiation effects within the treatment area and typically emerges weeks to months after therapy. On MRI, both ARE and tumor progression appear as enlarging enhancing regions on T1c imaging, with increased vasogenic edema on T2‐FLAIR imaging. This makes differentiating between these two conditions challenging in the clinic. The treatment approaches for ARE and tumor progression are quite different,^11^ highlighting the need for accurate diagnosis. Current diagnostic approaches for ARE rely on serial MRI scans, incorporating T1c, T2‐FLAIR, and perfusion imaging. When clinically feasible, a biopsy is performed to confirm the diagnosis histologically.^10^, ^12^, ^13^

Manual assessment of radiotherapy response in BM is time‐consuming and resource‐intensive in the clinic. For precise assessment, expert clinicians should accurately delineate and longitudinally compare multiple lesions on several volumetric images within a serial MRI to detect subtle changes in tumor sizes. This task is demanding, as tumors often exhibit complex and heterogeneous appearances, with varying patterns of growth and shrinkage, particularly after the radiation treatment. These challenges underscore the need for automated frameworks for longitudinal tumor segmentation and radiotherapy outcome assessment on serial MRI to improve and streamline the neuro‐oncology workflow in the management of BMs.

Developing automated models for precise segmentation of brain tumors has gained much research effort during recent years. Convolutional neural networks (CNNs) have become fundamental in medical image analysis due to their ability to automatically extract complex features from imaging data.^14^, ^15^ Among deep learning models, 3D U‐Net has demonstrated strong performance in brain tumor segmentation by replacing 2D operations with 3D counterparts, improving volumetric analysis.^16^ Following this, several U‐Net variants have been developed to enhance segmentation accuracy. Examples include ResU‐Net^17^ which incorporates residual connections, and SA‐Net,^18^ which introduces scale attention mechanisms. Other models, including AGSE‐VNet^19^ and SE‐NL V‐Net,^20^ integrate attention and squeeze‐and‐excitation modules to improve segmentation robustness. Additionally, cascaded U‐Net^21^ has demonstrated effective multi‐stage processing for refined segmentation. Among U‐Net variants, the nnU‐Net model^22^ stands out for its adaptability to new datasets and automatic configuration selection. The 3D U‐Net architecture has been adapted in a recently proposed framework for automatic tumor segmentation and radiotherapy outcome assessment with promising results in longitudinal tumor evaluation.^23^ However, U‐Net and its variants face limitations, particularly due to the restricted kernel sizes in convolutional layers, which hinder their ability to model long‐range dependencies, potentially affecting segmentation performance for tumors of varying sizes.^24^ Recent studies have investigated transformer architecture to address the issue of capturing long‐range dependencies in image segmentation. Models like TransBTS,^25^ UNETR,^26^ and Swin UNETR^27^ combine CNNs with transformers to leverage both local spatial features and global contextual information to improve segmentation accuracy. While previous studies have made notable progress in brain tumor segmentation, they often struggle with accurately delineating smaller tumors, despite showing good performance on medium sized to large tumors.^28^, ^29^, ^30^ Precise segmentation of smaller tumors is essential for effective radiotherapy planning and for evaluating post‐treatment responses, particularly when tumors shrink during treatment.

In this study, we propose a multi‐step deep learning framework for the automated segmentation and radiotherapy outcome assessment in BMs. The framework is designed to address the challenge of accurately delineating and longitudinally tracking small BMs, which are frequently overlooked by existing segmentation methods. A two‐stage strategy is adopted in the framework, where whole‐brain MRI scans are initially processed to localize the BMs, followed by analyzing tumor‐centered sub‐volumes at high resolution for precise tumor delineation. This two‐stage strategy preserves global coverage while dedicating focused processing to each lesion, where tumor probability maps from the first stage serve as priors to guide the refined segmentation. The framework incorporates new transformer blocks where the conventional window‐based self‐attention has been replaced with the neighborhood attention mechanism^31^ that we extended to 3D to capture subtle local context around each voxel while maintaining multi‐scale representation at different stages of encoder. This mechanism enhances local context modeling and enables the network to learn fine boundary cues and intensity variations, which are critical for segmentation of smaller lesions. The combination of the two‐stage processing strategy, probability map guidance, and 3D neighborhood attention allows the model to remain sensitive to small lesions while retaining the global context needed for accurate segmentation across different tumor sizes. Building on this foundation, we present an automated outcome assessment module that tracks each tumor across the baseline and several follow‐up MRIs, quantifies longitudinal tumor size changes, determines lesion status at each scan, and evaluates radiotherapy response based on the RANO‐BM criteria. The proposed framework was evaluated across different tumor size categories using an independent external test set. The results demonstrate that the framework outperforms existing segmentation models across tumors of various sizes, ranging from 2 mm to 5.6 cm.

## 2 MATERIALS AND METHODS

### 2.1 Data acquisition and preparation

The imaging data for development, optimization, and independent evaluation of the proposed framework were acquired from the BraTS^32^ and BraTS‐METS^33^ datasets, in addition to a study conducted at Sunnybrook Health Sciences Centre (SHSC) in Toronto, Canada, following the institutional research ethics board approval.

The BraTS^32^ and BraTS‐METS^33^ datasets include imaging data from 1131 glioma patients (6545 lesions) and 402 patients with brain metastasis (3076 lesions), respectively. Both datasets include co‐registered T1c and T2‐FLAIR images with a size of 240 × 240 × 155 voxels, as well as the ground‐truth masks for enhancing tumor and non‐enhancing (necrotic) tumor core. All images were resampled to a size of 512 × 512 × 200 voxels before processing by the framework. The ground‐truth tumor masks were generated by performing a union operation on the enhancing and non‐enhancing tumor masks. The BraTS dataset was used for model pre‐training. The BraTS‐METS dataset was split at the patient level into the training (70%, 280 patients, 2139 tumors), validation (5%, 22 patients, 154 tumors), and internal test (25%, 100 patients, 783 tumors) sets. The model training experiments were repeated three times using different random splits of the training and validation sets. The internal test set was kept entirely independent and unseen during all rounds of model training and optimization.

The data obtained from the study conducted at SHSC was used exclusively as an external test set for the independent evaluation of models in automated tumor segmentation and radiotherapy outcome assessment. In that study, the imaging and clinical data were collected from 212 patients (508 lesions) diagnosed with BM and treated with hypo‐fractionated SRS. Cystic lesions and those with prior resection or radiotherapy were excluded from the study. The study cohort consisted of 91 male (36.7%) and 121 female (63.3%) patients. The tumors had an average size of 1.7 ± 0.9 cm (range: 0.2–5.6 cm) at the baseline. The primary tumor histology included lung cancer (206 tumors, 40.6%), breast cancer (146 tumors, 28.7%), skin malignancies (60 tumors, 11.8%), renal cell carcinoma (25 tumors, 4.9%), esophageal cancer (22 tumors, 4.3%), colorectal cancer (21 tumors, 4.1%), and other cancers (28 tumors, 5.6%). Imaging data included T1c and T2‐FLAIR images acquired as part of routine clinical care for BM patients. These images were obtained prior to SRS (baseline) for treatment planning and at up to six follow‐up visits on a 2–6‐month schedule for standard therapy outcome assessment. The majority of the images had a size of 512 × 512 × 200 voxels, with an in‐plane resolution of 0.5 × 0.5 mm, and a slice thickness of 1 mm. Some T1c and T2‐FLAIR images had a size of 480 × 480 × 200 voxels and 448 × 448 × 139 voxels, respectively. All images were resampled to a size of 512 × 512 × 200 voxels. For each imaging session, the T2‐FLAIR image was co‐registered to the T1c volume using rigid registration. Skull stripping was performed using the HD‐BET algorithm.^34^

The gross tumor volumes (GTVs) on baseline imaging were delineated by a specialist in neuro‐radiation oncology and subsequently reviewed by at least one other neuro‐radiation oncologist and/or a neuro‐radiologist. The GTV contours were used to create tumor masks, which served as the ground truth in this study. The tumors were monitored after the SRS on serial follow‐up MRI by a neuro‐radiation oncologist to assess tumor size dynamics and determine the therapy outcome. The longest diameter of each tumor at the baseline and each follow‐up scan was measured and recorded. The clinical tumor status at each follow‐up scan (shrinkage [PR/CR], steady [SD], and enlargement [PD]), as well as the therapy outcome (LC/LF) was determined based on the RANO‐BM criteria,^9^ and served as the ground truth in this study. ARE was diagnosed and differentiated from tumor progression clinicoradiologically based on serial imaging.^10^

### 2.2 Framework architecture

**FIGURE 1:**
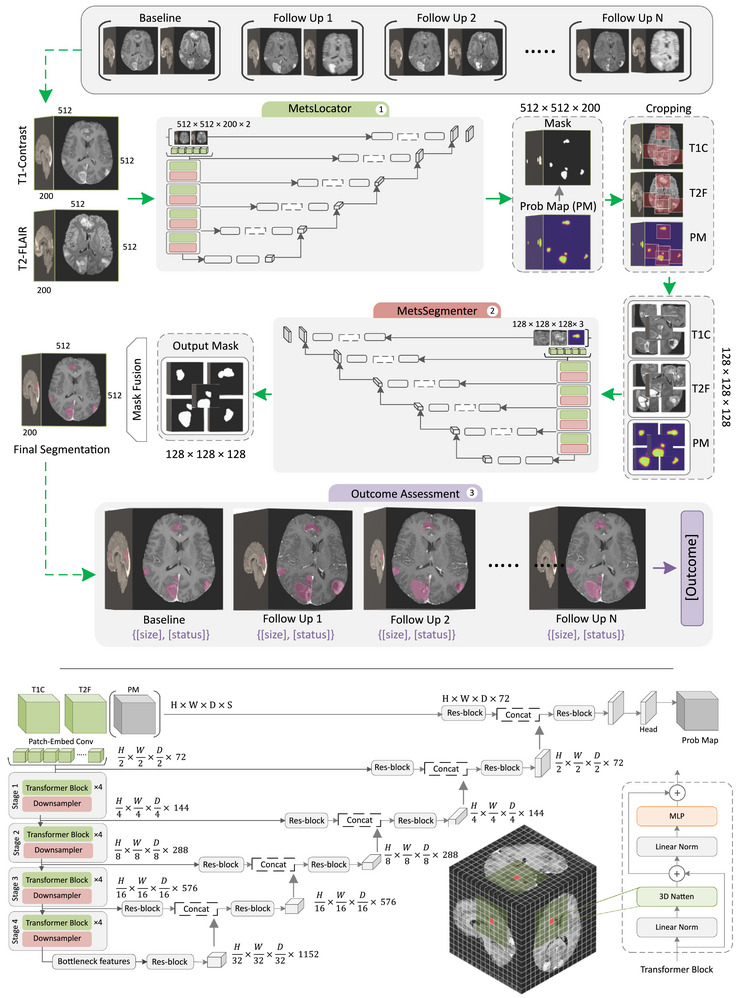
Overview of the proposed framework for tumor segmentation and radiotherapy outcome assessment on serial MRI. The 3D T1c and T2‐FLAIR images (size: 512 × 512 × 200 voxels) acquired at each imaging session are initially analyzed by the MetsLocator to identify potential metastatic tumor regions. Tumor‐centered volumes of size 128 × 128 × 128 voxels are then cropped from the 3D images and probability maps and analyzed by the MetsSegmenter for precise segmentation of the metastatic lesions. The segmentation masks generated for these cropped volumes are fused together to reconstruct a full segmentation mask of size 512 × 512 × 200 voxels. Finally, the segmentation masks obtained at the baseline and follow‐up sessions are passed to the outcome assessment module for evaluating longitudinal tumor size dynamics. The lower portion of the figure illustrates the inner architecture of the networks. The encoder utilizes a 3D transformer with neighborhood attention (Natten) to extract multi‐scale features from the multi‐modal magnetic resonance imaging (MRI) input at different stages. These features are passed through residual blocks and skip connections to a convolutional neural networks (CNN)‐based decoder to generate the segmentation probability map.

Figure 1 presents a schematic of the proposed framework designed for the automated localization, segmentation, and automated radiotherapy outcome assessment of brain metastases, using 3D serial MRI. The framework consists of two cascaded networks, namely, MetsLocator and MetsSegmenter, followed by an outcome assessment module. The MetsLocator identifies the tumor locations, while the MetsSegmenter performs precise segmentation on the localized metastases. The input to the MetsLocator included 3D T1c and T2‐FLAIR images with a size of 512 × 512 × 200 voxels. It generates a tumor probability map, which is binarized using a threshold of 0.5 to identify the presence and approximate locations of metastatic tumors. Once a tumor is detected, the binary mask is used to extract a region of interest (ROI) centered around the metastasis, cropping the T1c and T2‐FLAIR volumes to a standardized size of 128 × 128 × 128 voxels. If multiple tumors are present, the framework processes them independently by extracting a separate ROI for each lesion. For each follow‐up session, the tumors localized by the MetsLocator are cross‐checked with those of the previous scan. If a tumor from the previous scan is not localized in the current scan, a cropping is performed based on the previous coordinates and the cropped volumes are passed to the MetsSegmenter for further analysis. This cross‐checking is applied to differentiate between total disappearance (CR) of a tumor after SRS and substantial shrinkage to a very small size (PR).

The cropped volumes, along with the corresponding probability map, serve as the input to the MetsSegmenter network, delineating the tumor boundaries precisely within the cropped volume. The segmentation masks of size 128 × 128 × 128 voxels generated for the individual tumors are then fused based on their spatial coordinates in the original MRI volume to reconstruct a segmentation mask of size 512 × 512 × 200 voxels associated with all tumors. The longitudinal segmentation masks generated for each patient are then passed to the outcome assessment module, where changes in each lesion size and status are analyzed across the baseline and follow‐up scans to determine the radiotherapy outcome (described in Section 2.5).

A U‐shaped transformer‐based encoder/decoder architecture similar to Swin UNETR^27^ was adapted for the MetsLocator and MetsSegmenter networks, as shown in Figure 1. A 3D neighborhood self‐attention mechanism was integrated in the transformer blocks of this architecture, replacing the conventional window multi‐head self‐attention (WMSA) and shifted window multi‐head self‐attention (SWMSA) blocks.

In the encoder, the input volume is processed as a 3D sub‐volume $X\in R^{H\times W\times D\times S}$ with patches of size $H^{\prime }\times W^{\prime }\times D^{\prime }\times S=2\times 2\times 2\times S$, where *S* is the number of input channels (2 and 3 in the MetsLocator and MetsSegmenter, respectively). A patch embedding convolutional layer generates a sequence of 3D tokens with dimensions $\lceil \frac{H}{H^{\prime }}\rceil \times \lceil \frac{W}{W^{\prime }}\rceil \times \;\lceil \frac{D}{D^{\prime }}\rceil =H^{\circ }\times W^{\circ }\times D^{\circ }$ which are projected into an embedding space *E = 72*. The encoder backbone consists of four stages, each with multiple transformer blocks, followed by a down‐sampler. Within each transformer block, the 3D neighborhood attention (Natten) module computes self‐attention within local neighborhoods while maintaining translational equivariance. Unlike the WMSA and SWMSA mechanisms, the Natten operates over a sliding local neighborhood of size M×M×M=7×7×7, so that for each token location (i,j,k) the query (*Q*), key (*K*), and value (*V*) vectors are derived for the feature vectors within the neighborhood. The attention scores are then computed using a dot product between the central query and each of its *n* neighboring keys, with an added learnable relative position bias. Specifically, the attention score matrix $A_{ijk}\in R^{n\times 1}$ for a central token at position (*i*, *j*, *k*) is defined as:

(1) $$\;A_{ijk}=\left(\begin{array}{c} Q_{ijk}K_{t_{1}\left(i,j,k\right)}^{T}+B_{\left(ijk,t_{1}\left(i,j,k\right)\right)}\\ Q_{ijk}K_{t_{2}\left(i,j,k\right)}^{T}+B_{\left(ijk,t_{2}\left(i,j,k\right)\right)}\\ \vdots \\ Q_{ijk}K_{t_{n}\left(i,j,k\right)}^{T}+B_{\left(ijk,t_{n}\left(i,j,k\right)\right)} \end{array}\right)\;$$

where $\left\{t_{1}\left(i,j,k\right),t_{2}\left(i,j,k\right),\text{…}t_{n}\left(i,j,k\right)\right\}$ are the indices of the *n* neighbors of the token (*i*, *j*, *k*), and *B* is the learnable relative position bias. The corresponding value matrix $V_{ijk}$ is formed by stacking the value vectors of the neighbors:

(2) $$\;V_{ijk}={\left[\;V_{t_{1}\left(i,j,k\right)}^{T}\;V_{t_{2}\left(i,j,k\right)}^{T}\;\cdots \;V_{t_{n}\left(i,j,k\right)}^{T}\;\right]}^{T}\;$$

The scores are normalized using the Softmax function to produce attention weights, which are then used to compute a weighted sum of the value vectors, resulting in the neighborhood attention output for token (*i*, *j*, *k*):

(3) $$NA\;\left(ijk\right)=\;\mathrm{softmax}\left(\frac{A_{ijk}}{\sqrt{d}}\right)\;V_{ijk}$$

here, *d* is the dimensionality of each token embedding, used to scale the dot‐product scores. This sliding mechanism preserves translational equivariance, efficiently captures local context, and naturally expands the receptive field without any window shifts or extra partitioning. It attends over every voxel's local neighborhood at different resolutions, ensuring that subtle boundary cues of lesions of various sizes are directly incorporated into the corresponding token representation. As a result, details of tumor boundaries remain intact, even for tiny tumors, while sufficient surrounding context is aggregated for accurate segmentation. At the end of each encoder stage, A down‐sampling layer with 3 × 3 × 3 convolution kernels, a stride of 2, and padding of 1 reduces the spatial dimension by a factor two, double the size of embedding space, and introduces overlapping receptive fields to smooth feature transitions before entering the next stage.

In the decoder, the feature maps of size $\left\lceil \frac{H}{2^{\left(t+1\right)}}\rceil \times \lceil \frac{W}{2^{\left(t+1\right)}}\rceil \times \lceil \frac{D}{2^{\left(t+1\right)}}\right\rceil$ from the encoder stage *t* (t∈−1,0,1,2,3,4) are forwarded into a residual block consisting of 3×3×3 depth‐wise convolutional layers to capture spatially localized and channel‐specific features. Feature maps are progressively upsampled using deconvolutional layers and concatenated with earlier‐stage feature maps followed by residual blocks. Finally, a segmentation head with a convolutional layer and Softmax activation generates 3D probability maps for segmentation. The model applies a voxel‐wise Dice loss function^35^ described by Equation 4:

(4) $$L\left(G,P\right)=1-\frac{2\sum_{i=1}^{I}G_{i}P_{i}}{\sum_{i=1}^{I}G_{i}^{2}+\sum_{i=1}^{I}P_{i}^{2}\;}$$

where *I* represent the number of voxels in the image, while $G_{i}$ and $P_{i}$ denote, respectively, the one‐hot encoded ground truth and the predicted probability at voxel i for being tumor.

### 2.3 Training configuration

All models were pre‐trained on the BraTS dataset for weight initialization, before training on the training subset of the BraTS‐METS dataset. The voxel intensities in each input image were normalized to have zero mean and unit standard deviation based on non‐zero voxels. To improve robustness of models to intensity and orientation variations among the input images, random intensity, and spatial augmentations were applied during model training. Model training was performed using two NVIDIA L40s GPUs, each with 46 GB of memory, for up to 300 epochs. Training followed a linear warm‐up phase and a cosine annealing learning rate schedule. The AdamW optimizer was used with an initial learning rate of 0.0001, weight decay regularization of 1 × 10^−5^, and a momentum of 0.99. The training process was distributed across the GPUs, and a batch size of 1 was set per GPU. Model performance was continuously monitored on the training and validation sets, and early stopping was applied based on validation loss to prevent overfitting.

### 2.4 Evaluation metrics and comparative models

The framework performance in tumor segmentation was evaluated on the unseen test subset of the BraTS‐METS dataset, as well as the independent external dataset acquired in SHSC. The evaluation metrics included the Dice similarity coefficient (DSC)^36^ to evaluate voxel‐wise overlap, Hausdorff distance (HD)^37^ to assess boundary accuracy, and the tumor volume estimation error (VEE)^38^ to quantify volumetric agreement between the ground‐truth masks and segmentation masks generated by the model. For the SHSC dataset, the longest diameter of tumors obtained from the 3D segmentation masks at the baseline and follow‐up scans were compared to those determined by the neuro‐radiation oncologist and the absolute estimation error was calculated. The evaluation was performed for all tumors, as well as the small (≤1 cm; *n* = 128), medium (>1 cm and ≤2 cm; *n* = 214), and large (>2 cm; *n* = 166) tumor size categories separately.

The performance of the proposed framework was benchmarked against three widely adopted baseline models that represent the current state‐of‐the‐art in medical image segmentation, including the 3D U‐Net,^39^ nnU‐Net,^22^ and the Swin UNETR.^27^ 3D U‐Net is a classical convolutional encoder–decoder model that serves as the foundation for many medical imaging pipelines. nnU‐Net is a self‐configuring framework that automatically adapts the processing and network configuration, including the input patch size, training batch size, and number of training epochs, to the dataset, establishing a standardized baseline across diverse challenges. Swin UNETR is a transformer‐based architecture that integrates hierarchical window‐based self‐attention within a U‐shaped encoder/decoder design. All baseline models were trained and evaluated following the same pre‐training, training, and evaluation protocols as the proposed framework.

To assess the contribution of different components of the proposed framework, ablation experiments were performed using four different model configurations. The first configuration evaluated the segmentation performance of the MetsLocator alone, demonstrating its ability to localize and generate preliminary tumor segmentation masks. In the second configuration, the transformer blocks in both the MetsLocator and MetsSegmenter networks included the WMSA and SWMSA mechanisms instead of the Natten. Also, the probability map generated by the MetsLocator was not applied as the third input channel of the MetsSegmenter. The third configuration incorporated the neighborhood attention mechanism in all transformer blocks, but the MetsSegmenter did not input the probability map generated by the MetsLocator. The fourth configuration represented the complete proposed framework, in which the Natten blocks were employed throughout and the MetsSegmenter received a three‐channel input including the probability map generated by the MetsLocator.

### 2.5 Automated outcome assessment

The framework performance in automated outcome assessment was evaluated using the independent external data acquired from the study conducted at SHSC. Following the clinical protocol, the longest diameter of each tumor was measured from the 3D segmentation masks generated by the model for the baseline and all follow‐up scans. The tumor status at each follow‐up scan was classified by comparing the tumor size against the baseline and nadir measurements. Following the RANO‐BM criteria,^9^ the tumor status at each follow‐up was categorized into: shrinkage (CR/PR) if there was a decrease of more than 30% in the longest diameter compared to the baseline measurement, steady (SD) if there was a decrease of less than 30% compared to the baseline but also less than 20% increase compared to the nadir, and enlargement (PD) if there was more that 20% increase in the longest diameter of tumor compared to the nadir. The automatically determined tumor statuses at all follow‐up scans were compared with the ground truth to evaluate the framework performance in terms of accuracy, precision, and recall. This evaluation aimed to systematically scrutinize the framework performance in estimating tumor size changes over time, and its capability in distinguishing tumor status at each follow‐up scan, compared to expert annotations.

The LC/LF and ARE outcome after SRS were automatically detected for each tumor by analyzing the pattern of tumor size changes on serial follow‐up imaging. Tumors demonstrating a sequence of shrinkage or stable statuses at follow‐up scans, with no enlargement observed, were classified with an outcome of LC. When an enlargement was detected for a tumor, the change in the tumor size at the next follow‐up scan was calculated compared to the scan in which the enlargement was detected. If the tumor size increased again (>1 mm to account for measurement errors) after an initial enlargement status, the tumor was classified with an LF outcome. Tumors that initially enlarged but decreased or remained stable in size at subsequent follow‐up scans were classified as LC with ARE. Since a tumor with ARE could possibly progress later and classified as LF, the classification of LC/LF and ARE outcomes were performed independently for each tumor. The automatically detected tumor outcomes were compared with the ground truth to evaluate the framework performance in terms of accuracy, sensitivity, and specificity. Kaplan–Meier analyses were performed to compare the time to LF and ARE events detected based on the clinical outcome assessment (ground truth) and the automated assessment performed by framework. A log‐rank test was used to identify statistically significant differences between the curves for each event.

## 3 RESULTS

**FIGURE 2:**
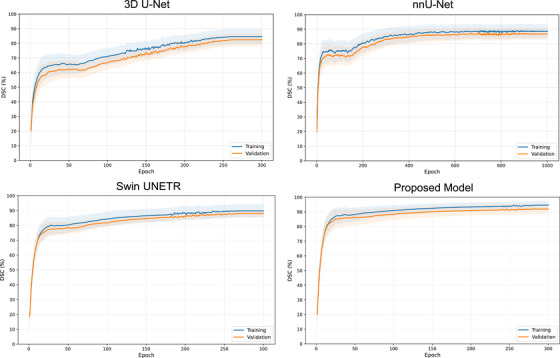
Training and validation Dice similarity coefficient (DSC) curves for the proposed and benchmarked models.

Figure 2 presents the training and validation DSC curves for the proposed and benchmarked models. All models exhibited stable and smooth convergence, with the proposed model achieving higher validation performance, implying improved generalization capability.

Table 1 presents the segmentation performance of the models on the test set of BraTS‐Mets dataset using different evaluation metrics. The results are further stratified based on the tumor size into three categories. This stratification aimed to compare how effectively each model delineates tumors of varying sizes, with a particular focus on the segmentation of smaller metastases (≤1 cm). Starting with the 3D U‐Net, the model achieved a DSC of 85.2 ± 7.2% and 79.4 ± 5.3% for large (>2 cm) and medium‐size (>1 cm and ≤2 cm) tumors, respectively. However, its performance dropped noticeably for small metastases (≤ 1 cm), with a DSC of 69.9 ± 8.4%. Similarly, nnU‐Net and Swin UNETR showed strong performance for larger tumors, with DSC scores of 87.4 ± 4.7% and 94.8 ± 2.9%, respectively. Their performance, however, decreased for small metastases, with DSC scores of 78.5 ± 5.9% for nnU‐Net and 84.2 ± 3.5% for Swin UNETR. Turning to ablated configurations of the proposed framework, the MetsLocator achieved a DSC of 86.0 ± 2.7% for small metastases. The framework configuration without the Natten mechanism and the probability map channel yielded a DSC of 87.8 ± 4.4% for small tumors. The third configuration that incorporated the Natten mechanism, but without the probability map channel, achieved a DSC of 89.6 ± 3.2% for small metastases. Finally, the complete framework resulted in the highest DSC of 91.4 ± 2.7% for small tumors. The results demonstrate that the proposed framework could effectively locate and delineate the tumors across all size categories, outperforming state‐of‐the‐art models, with a notable improvement in segmenting smaller tumors.

**TABLE 1:** Performance comparison of the segmentation models on the BraTS‐METS test set.

Model	Metric	All tumors	Tumor size ≤ 1 cm	1 cm < Tumor size ≤ 2 cm	Tumor size > 2 cm
3D U‐Net	DSC (%)	78.2 ± 4.1	69.9 ± 8.4	79.4 ± 5.3	85.2 ± 7.2
	HD (mm)	4.0 ± 0.4	4.9 ± 0.8	3.8 ± 0.5	3.4 ± 0.7
	VEE (cc)	0. 8 ± 0.4	0.8 ± 0.8	0.8 ± 0.6	0.7 ± 0.8
nnU‐Net	DSC (%)	82.9 ± 2.8	78.5 ± 5.9	82.9 ± 3.4	87.4 ± 4.7
	HD (mm)	3.4 ± 0.3	3.7 ± 0.6	3.4 ± 0.5	3.1 ± 0.7
	VEE (cc)	0.7 ± 0.3	0.8 ± 0.4	0.7 ± 0.7	0.7 ± 0.5
Swin UNETR	DSC (%)	89.9 ± 1.6	84.2 ± 3.5	90.7 ± 1.8	94.8 ± 2.9
	HD (mm)	2.4 ± 0.3	3.1 ± 0.7	2.3 ± 0.3	1.7 ± 0.2
	VEE (cc)	0.7 ± 0.2	0.7 ± 0.5	0.7 ± 0.4	0.6 ± 0.4
MetsLocator	DSC (%)	90.0 ± 1.70	86.0 ± 2.7	90.7 ± 2.1	95.0 ± 1.1
	HD (mm)	2.2 ± 0.3	3.0 ± 0.9	2.1 ± 0.8	1.3 ± 0.7
	VEE (cc)	0.7 ± 0.3	0.7 ± 0.5	0.6 ± 0.5	0.6 ± 0.4
Proposed model (w/o Natten and PM channel)	DSC (%)	91.4 ± 1.5	87.8 ± 4.4	91.1 ± 0.3	95.3 ± 0.9
	HD (mm)	1.7 ± 0.3	3.0 ± 0.3	1.3 ± 0.6	0.7 ± 0.7
	VEE (cc)	0.6 ± 0.3	0.7 ± 0.8	0.6 ± 0.2	0.5 ± 0.6
Proposed model (w/o PM channel)	DSC (%)	92.0 ± 1.3	89.6 ± 3.2	91.1 ± 1.2	95.2 ± 1.6
	HD (mm)	1.8 ± 0.4	2.3 ± 0.7	1.3 ± 0.7	1.7 ± 0.4
	VEE (cc)	0.5 ± 0.3	0.6 ± 0.4	0.5 ± 0.6	0.4 ± 0.7
Proposed model	DSC (%)	93.2 ± 1.1	91.4 ± 2.7	92.4 ± 1.7	95.7 ± 1.2
	HD (mm)	1.8 ± 0.2	2.1 ± 0.5	1.8 ± 0.3	1.6 ± 0.4
	VEE (cc)	0.5 ± 0.3	0.5 ± 0.6	0.4 ± 0.4	0.4 ± 0.5

*Note*: The values indicate mean ± standard deviation.

Abbreviations: DSC, Dice similarity coefficient; HD, Hausdorff distance; Natten, neighborhood attention; PM, probability map; VEE, volume estimation error.

Table 2 presents the models performance on the external SHSC dataset across different categories of baseline tumor size. A similar performance trend was observed across different tumor size categories. While the 3D U‐Net, nnU‐Net, and Swin UNETR models demonstrated a relatively acceptable performance on the larger tumors, their performance dropped for small tumors, demonstrating a DSC of 64.2 ± 6.5%, 72.8 ± 6.4%, and 81.4 ± 6.1%, respectively. The MetsLocator achieved a DSC of 82.1 ± 4.8% for small tumors, while the other two ablated configurations of the proposed framework improved the DSC of these tumors to 84.8 ± 5.1% and 87.4 ± 3.7%, respectively. The complete framework achieved a DSC of 89.8 ± 3.4%, 92.0 ± 3.0%, and 93.1 ± 2.3% for the three tumor size categories, respectively, considerably outperforming the benchmarked models especially in segmenting small metastases.

**TABLE 2:** Performance comparison of the segmentation models on the external dataset acquired from SHSC.

Model	Metric	All tumors	Tumor size ≤ 1 cm	1 cm < Tumor size ≤ 2 cm	Tumor size > 2 cm
3D U‐Net	DSC (%)	73.0 ± 3.1	64.2 ± 6.5	73.9 ± 4.9	80.8 ± 4.8
	HD (mm)	5.4 ± 0.3	6.9 ± 0.4	5.3 ± 0.8	4.0 ± 0.3
	VEE (cc)	0.8 ± 0.3	0.9 ± 0.2	0.8 ± 0.3	0.8 ± 0.8
nnU‐Net	DSC (%)	77.3 ± 3.0	72.8 ± 6.4	76.2 ± 4.9	82.9 ± 3.9
	HD (mm)	5.5 ± 0.3	6.7 ± 0.5	5.0 ± 0.8	4.9 ± 0.4
	VEE (cc)	0.8 ± 0.4	0.8 ± 0.7	0.8 ± 0.6	0.8 ± 0.8
Swin UNETR	DSC (%)	84.3 ± 3.1	81.4 ± 6.1	84.5 ± 4.7	86.9 ± 5.1
	HD (mm)	3.8 ± 0.5	4.9 ± 0.5	3.9 ± 0.9	2.7 ± 0.9
	VEE (cc)	0.7 ± 0.4	0.8 ± 0.7	0.8 ± 0.2	0.7 ± 0.8
MetsLocator	DSC (%)	85.9 ± 2.6	82.1 ± 4.8	86.4 ± 4.3	87.1 ± 4.5
	HD (mm)	3.1 ± 0.5	4.1 ± 0.7	3.3 ± 0.5	2.1 ± 0.7
	VEE (cc)	0.7 ± 0.2	0.7 ± 0.5	0.7 ± 0.6	0.7 ± 0.4
Proposed model (w/o Natten and PM channel)	DSC (%)	87.2 ± 2.5	84.8 ± 5.1	88.1 ± 3.2	88.7 ± 4.3
	HD (mm)	2.5 ± 0.3	3.0 ± 0.7	3.0 ± 0.4	1.5 ± 0.4
	VEE (cc)	0. 7 ± 0.4	0.7 ± 0.4	0.7 ± 0.8	0.6 ± 0.8
Proposed model (w/o PM channel)	DSC (%)	90.2 ± 1.9	87.4 ± 3.7	90.8 ± 3.5	92.4 ± 2.5
	HD (mm)	2.1 ± 0.2	3.0 ± 0.5	1.8 ± 0.3	1.6 ± 0.3
	VEE (cc)	0.5 ± 0.4	0.6 ± 0.7	0.5 ± 0.4	0.3 ± 0.8
Proposed model	DSC (%)	91.6 ± 1.7	89.8 ± 3.4	92.0 ± 3.0	93.1 ± 2.3
	HD (mm)	1.8 ± 0.3	2.1 ± 0.6	1.8 ± 0.4	1.6 ± 0.5
	VEE (cc)	0.4 ± 0.4	0.5 ± 0.4	0.5 ± 0.9	0.3 ± 0.9

*Note*: Tumor size categories indicate baseline measurements. The values indicate mean ± standard deviation.

Abbreviations: DSC, Dice similarity coefficient; HD, Hausdorff distance; Natten, neighborhood attention; PM, probability map; SHSC, Sunnybrook Health Sciences Centre; VEE, volume estimation error.

**FIGURE 3:**
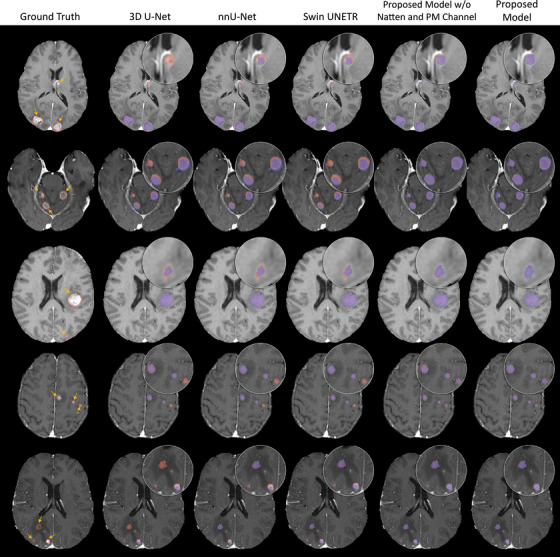
Qualitative comparison of models’ performance on Sunnybrook Health Sciences Centre (SHSC) tests set for five representative patients. In the first column, the yellow arrows indicate the tumor locations while the red contours represent the ground‐truth tumor boundary. In other columns, red, blue, and purple overlays represent the ground truth, segmentation mask generated by models, and their overlap, respectively.

Figure 3 presents the qualitative segmentation results on the SHSC dataset. Both 3D U‐Net and nnU‐Net exhibited under‐segmentation, especially for small tumors, often missing parts of the metastases. Similarly, the Swin UNETR performed relatively better for larger tumors but demonstrated limitations in capturing the boundaries of small metastases accurately. The proposed models, particularly when integrated with the Natten and probability map channel, notably outperformed the other models in precise segmentation of the tumors, and particularly the smaller ones.

Table 3 presents the average errors in estimating the longest diameter of tumor from the 3D segmentation masks generated by the models for the baseline and follow‐up scans of the external SHSC dataset. The error was calculated for each tumor using the longest diameter measured by the neuro‐radiation oncologist as the ground truth. The 3D U‐Net and nnU‐Net models demonstrated an average error of 3–5 mm in estimating the tumor size at the baseline and follow‐up scans, with larger errors associated with the smaller tumors. The Swin UNETR reduced the error to about 2–4 mm, with better performance on the large and medium size categories. The two ablated configurations of the proposed model further reduced the error to about 1–3 mm. The complete model, integrating both the Natten and parametric map channel, achieved the lowest baseline errors across all tumor sizes, with a mean error of 1.3  ±  0.9 mm for the small, 1.1  ±  0.9 mm for medium‐size, and 1.0  ±  0.7 mm for the large tumor categories. This model consistently maintained minimal errors across all follow‐ups, with mean errors of 1.1  ±  0.7 mm (small), 1.0 ±  0.7 mm (medium), and 1.0  ±  0.6 mm (large) at the fifth follow‐up scan, demonstrating the best performance among all evaluated methods.

**TABLE 3:** Average absolute errors in estimating the tumor longest diameter at the baseline and the follow‐up scans of the external SHSC dataset, based on the 3D segmentation masks generated by different models.

Model	Baseline	1st Follow‐up	2nd Follow‐up	3rd Follow‐up	4th Follow‐up	5th Follow‐up
	**Baseline tumor size ≤ 1 cm**					
3D U‐Net	4.6 ± 2.8 mm	4.1 ± 2.9 mm	4.0 ± 2.5 mm	3.7 ± 2.4 mm	3.6 ± 2.0 mm	3.1 ± 1.2 mm
nnU‐Net	4.0 ± 1.7 mm	3.9 ± 2.2 mm	3.1 ± 2.1 mm	3.4 ± 2.2 mm	3.1 ± 2.2 mm	3.0 ± 2.9 mm
Swin UNETR	3.5 ± 1.4 mm	3.0 ± 2.1 mm	2.8 ± 1.1 mm	3.3 ± 1.3 mm	3.0 ± 2.3 mm	2.7 ± 1.9 mm
Proposed model (w/o Natten and PM channel)	2.2 ± 1.5 mm	2.4 ± 1.6 mm	2.1 ± 1.5 mm	2.8 ± 1.1 mm	2.4 ± 1.1 mm	2.2 ± 1.1 mm
Proposed model (w/o PM channel)	1.7 ± 1.1 mm	2.2 ± 1.1 mm	1.8 ± 1.0 mm	2.0 ± 0.9 mm	1.6 ± 1.0 mm	1.8 ± 1.0 mm
Proposed model (complete)	1.3 ± 0.9 mm	1.2 ± 0.9 mm	1.2 ± 0.7 mm	1.3 ± 0.7 mm	1.2 ± 0.8 mm	1.1 ± 0.7 mm
	**1 cm < Baseline tumor size ≤ 2 cm**					
3D U‐Net	4.2 ± 2.2 mm	4.0 ± 2.1 mm	3.4 ± 1.8 mm	3.6 ± 2.3 mm	3.3 ± 2.6 mm	3.1 ± 2.6 mm
nnU‐Net	3.9 ± 1.9 mm	3.9 ± 1.2 mm	3.0 ± 1.3 mm	3.2 ± 2.0 mm	3.0 ± 1.9 mm	2.9 ± 1.6 mm
Swin UNETR	3.0 ± 1.7 mm	2.9 ± 1.7 mm	2.5 ± 1.4 mm	2.6 ± 1.1 mm	2.5 ± 1.3 mm	2.7 ± 1.8 mm
Proposed model (w/o Natten and PM channel)	2.2 ± 1.3 mm	2.4 ± 1.9 mm	2.1 ± 1.7 mm	2.0 ± 1.2 mm	1.8 ± 1.0 mm	2.0 ± 1.1 mm
Proposed model (w/o PM channel)	1.5 ± 1.1 mm	1.2 ± 1.1 mm	1.1 ± 1.0 mm	1.5 ± 1.0 mm	1.4 ± 1.0 mm	1.5 ± 0.9 mm
Proposed model (complete)	1.1 ± 0.9 mm	1.0 ± 0.6 mm	1.0 ± 0.3 mm	1.1 ± 1.0 mm	1.1 ± 0.7 mm	1.0 ± 0.7 mm
	**Baseline tumor size > 2 cm**					
3D U‐Net	3.9 ± 1.8 mm	3.1 ± 1.9 mm	3.0 ± 2.0 mm	3.0 ± 2.0 mm	3.0 ± 2.0 mm	2.9 ± 1.9 mm
nnU‐Net	3.8 ± 1.1 mm	2.9 ± 1.9 mm	2.8 ± 1.7 mm	2.8 ± 1.1 mm	3.1 ± 2.1 mm	2.9 ± 1.2 mm
Swin UNETR	2.0 ± 1.0 mm	2.1 ± 1.5 mm	2.1 ± 1.8 mm	2.2 ± 1.9 mm	2.0 ± 1.8 mm	2.2 ± 1.5 mm
Proposed model (w/o Natten and PM channel)	1.2 ± 1.0 mm	1.5 ± 1.0 mm	1.3 ± 1.2 mm	1.3 ± 1.0 mm	1.5 ± 0.7 mm	1.6 ± 1.1 mm
Proposed model (w/o PM channel)	1.2 ± 0.9 mm	1.5 ± 1.1 mm	1.3 ± 1.0 mm	1.3 ± 0.9 mm	1.2 ± 1.0 mm	1.4 ± 0.7 mm
Proposed model (complete)	1.0 ± 0.7 mm	1.0 ± 0.9 mm	1.0 ± 0.8 mm	1.0 ± 0.3 mm	1.1 ± 0.5 mm	1.0 ± 0.6 mm

*Note*: The values indicate mean ± standard deviation.

Abbreviations: Natten, neighborhood attention; PM, probability map; SHSC, Sunnybrook Health Sciences Centre.

Table 4 presents the performance of different models on the SHSC dataset in detecting tumor status (shrinkage, steady, and enlargement) at follow‐up scans after SRS. The 3D U‐Net showed a modest performance for the small and medium size categories, with an accuracy of 80.6 ± 1.5% and 81.5 ± 1.5%, respectively. Its performance improved for larger tumors, where the accuracy reached 83.2 ± 1.9%. The nnU‐Net performed better than 3D U‐Net across all tumor sizes, with an accuracy of 83.2 ± 1.1%, 85.2 ± 0.5%, and 87.4 ± 1.7% for the small, medium‐size, and large tumor categories, respectively. The Swin UNETR improved these accuracies to 86.7 ± 0.8%, 88.0 ± 1.9%, and 89.2 ± 1.1%, respectively. The ablated configurations of the proposed model outperformed the previous models with accuracies of 87.9 ± 0.3% and 89.8 ± 0.9% for small, 89.2 ± 0.9% and 91.2 ± 0.7% for medium‐size, and 90.4 ± 1.4% and 92.1 ± 0.6% for large tumors, respectively. The complete model delivered the best overall performance for all tumor size categories. It reached the accuracy of 91.0 ± 0.5% for the small, 92.5 ± 0.8% for medium‐size, and 93.8 ± 0.5% for the large tumors, with highest precisions and recalls across the board.

**TABLE 4:** Performance of different models on the external SHSC dataset in detecting tumor status at the follow‐up scans after SRS, based on the RANO‐BM criteria.

		Baseline tumor size ≤1 cm			1 cm < Baseline tumor size ≤ 2 cm			Baseline tumor size > 2 cm		
Model	Tumor size status	Accuracy (%)	Precision (%)	Recall (%)	Accuracy (%)	Precision (%)	Recall (%)	Accuracy (%)	Precision (%)	Recall (%)
3D U‐Net	Shrinkage	80.6 ± 1.5	78.6 ± 1.3	79.8 ± 1.0	81.5 ± 1.5	79.8 ± 1.5	80.0 ± 1.3	83.2 ± 1.9	82.9 ± 1.9	83.0 ± 1.2
	Steady		84.0 ± 0.5	82.5 ± 1.4		81.5 ± 1.1	79.9 ± 1.8		81.4 ± 1.7	81.8 ± 1.8
	Enlargement		82.9 ± 1.2	79.8 ± 1.7		85.1 ± 1.6	83.0 ± 1.5		86.8 ± 1.5	84.2 ± 0.5
nnU‐Net	Shrinkage	83.2 ± 1.1	82.8 ± 1.2	81.4 ± 1.0	85.2 ± 0.5	84.2 ± 0.8	84.9 ± 1.1	87.4 ± 1.7	88.5 ± 1.2	86.8 ± 1.2
	Steady		84.1 ± 1.5	84.2 ± 0.9		84.2 ± 1.0	85.0 ± 0.5		87.9 ± 0.9	87.5 ± 1.4
	Enlargement		84.7 ± 1.0	81.9 ± 1.1		87.5 ± 0.5	85.4 ± 0.9		88.4 ± 0.8	86.9 ± 1.1
Swin UNETR	Shrinkage	86.7 ± 0.8	84.5 ± 0.8	84.9 ± 1.0	88.0 ± 1.9	86.8 ± 1.1	85.2 ± 0.7	89.2 ± 1.1	91.2 ± 0.5	87.6 ± 1.5
	Steady		85.9 ± 0.5	86.5 ± 0.9		86.2 ± 0.9	86.4 ± 1.0		88.9 ± 1.5	89.5 ± 0.2
	Enlargement		87.2 ± 0.6	83.8 ± 1.1		89.4 ± 0.6	86.8 ± 0.5		90.1 ± 0.7	89.5 ± 1.0
Proposed model (w/o Natten and PM channel)	Shrinkage	87.9 ± 0.3	86.5 ± 0.8	85.9 ± 0.7	89.2 ± 0.9	89.2 ± 0.5	86.5 ± 0.4	90.4 ± 1.4	93.1 ± 1.2	89.9 ± 1.6
	Steady		88.2 ± 0.7	87.2 ± 0.6		88.8 ± 0.8	89.6 ± 1.1		92.9 ± 0.5	91.1 ± 1.0
	Enlargement		89.9 ± 1.0	84.6 ± 0.5		92.9 ± 0.8	87.5 ± 0.5		93.8 ± 1.0	90.4 ± 1.1
Proposed model (w/o PM channel)	Shrinkage	89.8 ± 0.9	88.1 ± 0.6	88.7 ± 0.3	91.2 ± 0.7	92.6 ± 1.1	88.7 ± 0.7	92.1 ± 0.6	94.4 ± 0.6	91.2 ± 0.8
	Steady		91.2 ± 0.7	90.0 ± 0.8		91.3 ± 0.5	91.5 ± 1.7		94.9 ± 0.4	92.0 ± 0.2
	Enlargement		92.6 ± 0.8	87.8 ± 0.4		94.0 ± 0.7	88.5 ± 0.7		96.0 ± 0.2	92.1 ± 0.4
Proposed model (complete)	Shrinkage	91.0 ± 0.5	90.0 ± 0.7	89.1 ± 0.8	92.5 ± 0.8	95.7 ± 0.3	89.4 ± 0.5	93.8 ± 0.5	96.5 ± 0.3	91.7 ± 0.2
	Steady		92.0 ± 0.8	92.3 ± 0.4		93.9 ± 0.7	94.5 ± 0.3		95.3 ± 0.8	95.8 ± 0.5
	Enlargement		93.0 ± 0.3	88.0 ± 0.6		96.2 ± 0.4	90.2 ± 0.8		97.0 ± 0.4	93.8 ± 0.8

*Note*: The values indicate mean ± standard deviation.

Abbreviations: Natten, neighborhood attention; PM, probability map; RANO‐BM, response assessment in neuro‐oncology brain metastases; SHSC, Sunnybrook Health Sciences Centre; SRS, stereotactic radiosurgery.

**FIGURE 4:**
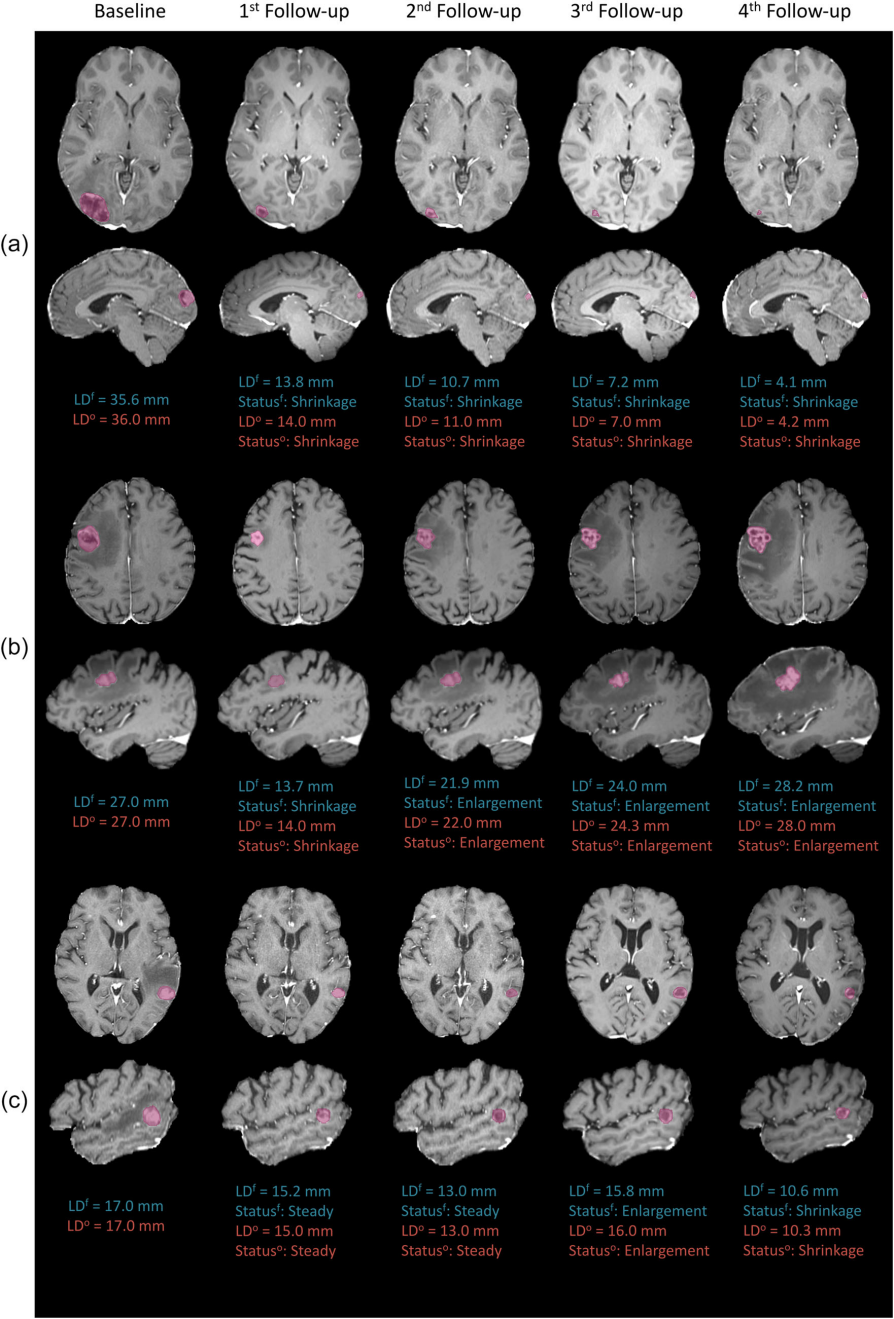
Serial T1c images acquired at the baseline and follow‐up scans after SRS from three representative patients with BM, demonstrating an outcome of: (a) LC, (b) LF detected at the 3rd follow‐up scan, and (c) ARE detected at the 4th follow‐up scan. The tumor longest diameter and status identified by the proposed framework (LD^f^, Status^f^) from the 3D segmentation masks, and by the neuro‐radiation oncologist (LD°, Status^o^) are shown for each scan. ARE, adverse radiation effect; BM; brain metastases; LC, local control; LF, local failure; Natten, neighborhood attention; PM, probability map; SHSC, Sunnybrook Health Sciences Centre; SRS, stereotactic radiosurgery.

Figure 4 presents the serial MRI acquired from three representative patients, with the tumor sizes and statuses at each scan identified by the proposed framework, versus those determined by the neuro‐radiation oncologist. The tumor in Figure 4a demonstrates a sequence of shrinkage status in all follow up scans and has been classified with an LC outcome by the framework and the oncologist. Figure 4b shows a tumor with shrinkage status in the first follow up, but an enlargement status in the second follow up, followed by further increase in size in the third follow up. This tumor has been classified with an LF outcome detected at the third follow up. The tumor in Figure 4c demonstrates a steady status in the first and second follow ups, but an enlargement in the third follow up followed by a decrease in size in the fourth follow up. This tumor has been classified with an LC outcome but with ARE detected at the fourth follow up.

Table 5 presents the performance of the models for automatic outcome assessment in terms of LC/LF and ARE detection on the external SHSC dataset. The 3D U‐Net demonstrated comparatively lower performance overall, with accuracies of 77.6 ± 1.9% and 78.6 ± 1.0% for LC/LF and ARE detection in small tumors, respectively. Its performance improved for larger tumors, reaching an accuracy of 78.0 ± 2.0% and 84.1 ± 2.5% for LC/LF and ARE detection in tumors > 2 cm. The nnU‐Net performed better than 3D U‐Net across all tumor sizes. It achieved an accuracy of 81.0 ± 1.9% for LC/LF detection and 83.8 ± 1.0% ARE detection in small tumors, increasing to 83.4 ± 0.5% and 85.2 ± 1.5%, respectively, in the large tumor category. The Swin UNETR further improved performance, reaching 86.0 ± 2.0% and 87.5 ± 2.0% for LC/LF and ARE detection in small tumors, respectively, and achieving 88.2 ± 1.0% and 90.1 ± 0.5% in the large tumor category. The ablated configurations of the proposed model outperformed the previous models, with accuracies of 90.0 ± 1.0% and 93.0 ± 0.5% for LC/LF detection and 90.0 ± 1.0% and 93.5 ± 1.0% for ARE detection in small tumors. For larger tumors, the ablated models achieved accuracies of up to 92.5 ± 1.0% and 94.2 ± 0.5% for LC/LF detection and 96.8 ± 1.0% and 97.0 ± 0.2% for ARE detection. The complete proposed model demonstrated the highest overall performance, with LC/LF and ARE detection accuracies of 96.7 ± 0.0% and 96.6 ± 0.0% in small tumors, 96.8 ± 0.0 % and 98.4 ± 0.0% in medium‐sized tumors, and 97.3 ± 0.0% and 100.0 ± 0.0% in large tumors, respectively.

**TABLE 5:** Performance of different models on the external SHSC dataset in detecting LC/LF and ARE outcomes for the tumors treated with SRS.

	LC/LF detection			ARE detection		
Model	Accuracy (%)	Sensitivity (%)	Specificity (%)	Accuracy (%)	Sensitivity (%)	Specificity (%)
	**Baseline tumor size ≤ 1 cm**					
3D U‐Net	77.6 ± 1.9	79.1 ± 1.9	100.0 ± 0.0	78.6 ± 1.0	75.3 ± 2.0	80.3 ± 2.0
nnU‐Net	81.0 ± 1.9	80.4 ± 1.9	100.0 ± 0.0	83.8 ± 1.0	80.5 ± 1.0	85.7 ± 3.0
Swin UNETR	86.0 ± 2.0	86.5 ± 3.5	100.0 ± 0.0	87.5 ± 2.0	82.8 ± 1.5	90.0 ± 2.5
Proposed model (w/o Natten and PM channel)	90.0 ± 1.0	89.5 ± 1.5	100.0 ± 0.0	90.0 ± 1.0	90.0 ± 0.0	90.0 ± 1.0
Proposed model (w/o PM channel)	93.0 ± 0.5	93.5 ± 1.0	100.0 ± 0.0	93.5 ± 1.0	90.0 ± 0.0	95.0 ± 1.0
Proposed model (complete)	96.7 ± 0.0	96.5 ± 0.0	100.0 ± 0.0	96.6 ± 0.0	100.0 ± 0.0	95.0 ± 0.0
	**1 cm < Baseline tumor size ≤ 2 cm**					
3D U‐Net	77.8 ± 2.5	80.1 ± 1.5	67.3 ± 2.5	83.8 ± 1.5	77.5 ± 1.5	86.1 ± 2.3
nnU‐Net	82.3 ± 0.5	86.7 ± 2.5	75.0 ± 1.0	84.5 ± 2.5	82.3 ± 3.0	88.9 ± 3.1
Swin UNETR	87.5 ± 0.5	88.5 ± 1.5	75.5 ± 1.0	88.7 ± 3.2	83.4 ± 2.0	91.1 ± 3.5
Proposed model (w/o Natten and PM channel)	90.1 ± 0.5	90.5 ± 1.0	87.5 ± 0.0	95.5 ± 1.5	94.1 ± 0.0	95.5 ± 2.5
Proposed model (w/o PM channel)	93.1 ± 1.5	93.5 ± 1.5	87.5 ± 0.0	96.1 ± 1.0	94.1 ± 0.0	97.0 ± 1.0
Proposed model (complete)	96.8 ± 0.0	98.1 ± 0.0	87.5 ± 0.0	98.4 ± 0.0	94.1 ± 0.0	100.0 ± 0.0
	**Baseline tumor size > 2 cm**					
3D U‐Net	78.0 ± 2.0	81.4 ± 2.5	70.1 ± 1.2	84.1 ± 2.5	78.4 ± 1.1	86.5 ± 1.5
nnU‐Net	83.4 ± 0.5	85.1 ± 1.1	75.2 ± 1.5	85.2 ± 1.5	83.2 ± 2.2	90.0 ± 1.5
Swin UNETR	88.2 ± 1.0	88.1 ± 1.5	79.5 ± 2.5	90.1 ± 0.5	84.8 ± 1.5	93.0 ± 1.0
Proposed model (w/o Natten and PM channel)	92.5 ± 1.0	90.0 ± 1.5	83.3 ± 0.0	96.8 ± 1.0	87.2 ± 0.5	96.0 ± 1.5
Proposed model (w/o PM channel)	94.2 ± 0.5	96.5 ± 0.4	83.3 ± 0.0	97.0 ± 0.2	100.0 ± 0.0	96.2 ± 0.4
Proposed model (complete)	97.3 ± 0.0	100.0 ± 0.0	83.3 ± 0.0	100.0 ± 0.0	100.0 ± 0.0	100.0 ± 0.0

*Note*: The values indicate mean ± standard deviation.

Abbreviations: ARE, adverse radiation effect; LC/LF, local control/failure; Natten, neighborhood attention; PM, probability map; SHSC, Sunnybrook Health Sciences Centre; SRS, stereotactic radiosurgery.

**FIGURE 5:**
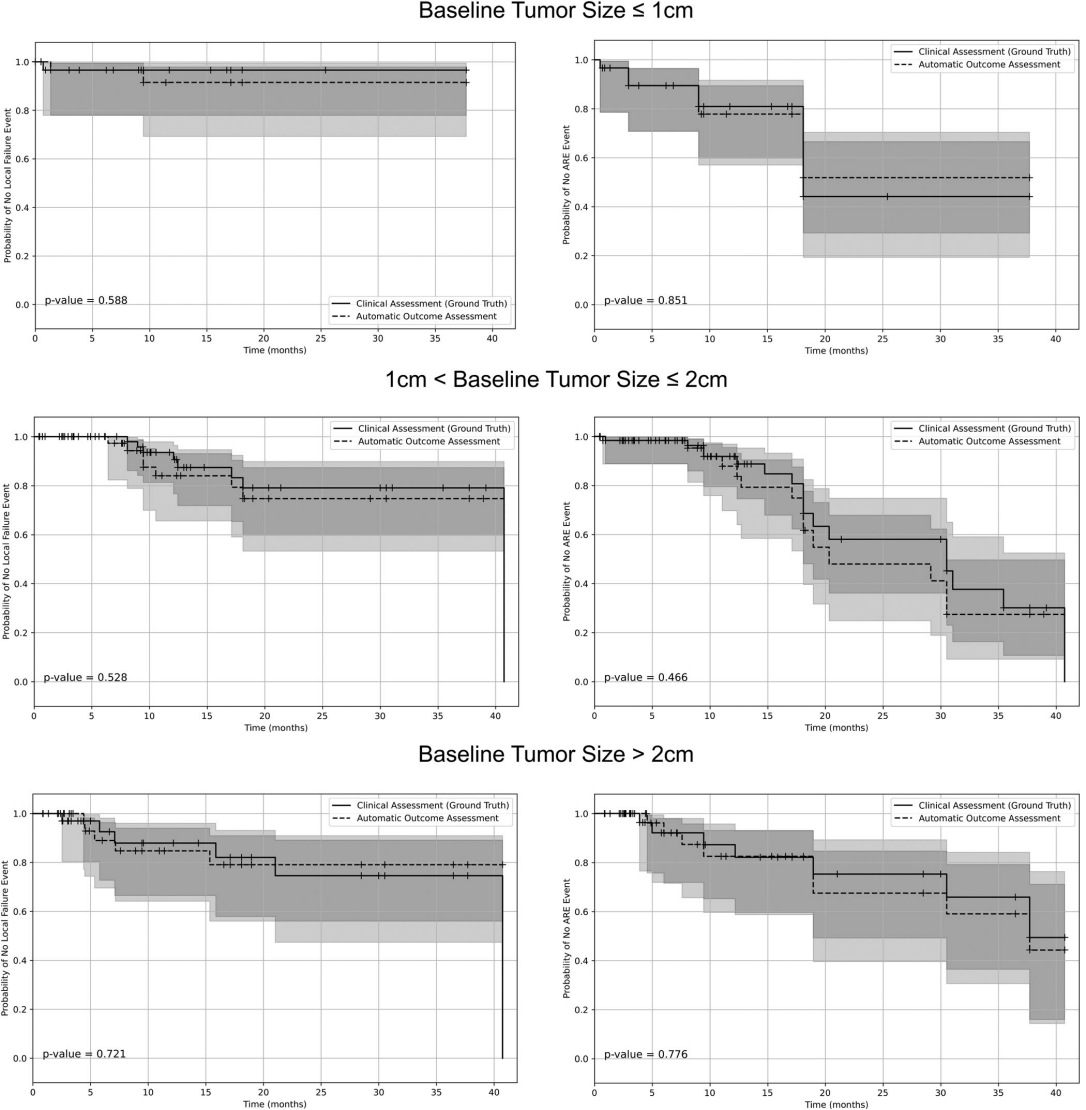
Kaplan–Meier curves for time‐to‐event analysis, comparing the clinical radiotherapy outcome assessment and the assessment performed by the proposed automated framework on the external SHSC dataset. Curves are stratified by the tumor sizes categories. The curves associated with the LF and ARE events are shown on the left and right columns, respectively. The time‐to‐event was calculated for each tumor from the radiotherapy date to the date an LF or ARE was detected. ARE, adverse radiation effect; LF, local failure; SHSC, Sunnybrook Health Sciences Centre.

Figure 5 shows the Kaplan–Meier curves for the time to LF and ARE events detected clinically and by the proposed automated framework. The curves associated with the proposed framework are similar to those obtained based on the clinical outcome assessment for all tumor size categories, with no statistically significant difference observed between the curves for the LF or ARE event. This similarity suggests that the proposed automated framework can reliably replicate clinical assessments in monitoring LF and ARE across different tumor sizes.

## 4 DISCUSSION AND CONCLUSION

In this study, an automated framework was presented for segmentation and radiotherapy outcome assessment of BM on standard serial MRI. The framework was designed to precisely segment and monitor longitudinal alterations in BM of all sizes, with a particular emphasis on smaller lesions. The framework was trained on the publicly available BraTS and BraTS‐METS datasets and independently evaluated on an external dataset acquired from BM patients treated with SRS, assessing its performance across different size categories of metastatic tumors. The framework was thoroughly investigated via multiple model configurations and benchmarked against state‐of‐the‐art segmentation models.

The results demonstrated that the proposed framework exhibits very good performance in longitudinal segmentation of brain metastases, even for small lesions. Notably, while all benchmarked models performed relatively well for larger tumors, they demonstrated a substantial drop in segmentation accuracy for smaller metastases. While the framework's performance on the external SHSC dataset was lower than on the BraTS‐METS test set, the difference was modest (∼2%) and expected given the out‐of‐distribution characteristics of the SHSC data. Specifically, BraTS‐METS is a curated research dataset with standardized distribution that was split in this study at patient level for model training, validation and testing. The SHSC data, however, represents a fully independent external dataset acquired with different scanners, imaging protocols, and resolutions. These factors naturally introduce domain variability in the data that is not fully eliminated by uniform preprocessing and intensity normalization. Despite this variability, the proposed framework maintained a strong performance on the SHSC dataset and consistently outperformed the baseline models on all tumor size categories, underscoring its robustness and generalizability to independent clinical data.

**FIGURE 6:**
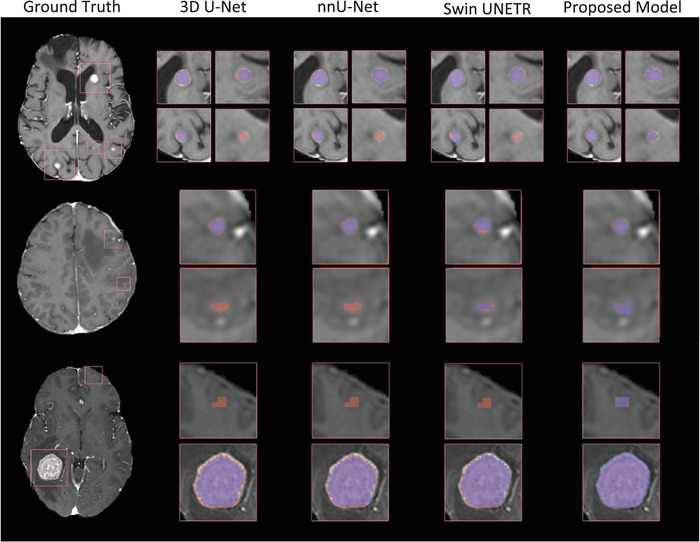
Representative examples of tumor segmentation errors associated with different models. In the first column, red boxes indicate lesion locations, and red contours represent the ground‐truth tumor boundaries. The subsequent columns show zoomed‐in views of each lesion, where red, blue, and purple overlays represent the ground truth, the segmentation mask generated by the model, and their overlap, respectively.

The tumor segmentation errors presented in Figure 6 for representative cases illustrate typical performance differences among the CNN‐based models (3D U‐Net and nnU‐Net), transformer‐based Swin UNETR, and the proposed framework. The CNN‐based models were typically unable to capture very small lesions, as fine‐grained signals tend to be suppressed through the pooling layers after the convolution with restricted kernel size, leading to missed detections in subtle cases. They also showed less precise delineation in tumors with complex morphology, reflecting the limitations of purely local feature extraction. Swin UNETR demonstrated improved detection and segmentation by leveraging the WMSA and SWMSA mechanisms to model long‐range dependencies, although its performance remained inconsistent, particularly for smaller lesions. In contrast, the proposed framework incorporating the cascaded processing strategy, tumor probability map guidance, and 3D Natten mechanism consistently localized and delineated metastases of various sizes, including lesions <5 mm, with only minor voxel‐level errors along the boundaries. These small discrepancies with the ground‐truth masks are comparable to the contouring variabilities generally observed between expert human annotators.

The proposed framework also showed very good performance in monitoring tumor size changes after SRS, identifying tumor size status in terms of response categories at individual follow‐up scans, and subsequently in assessing the radiotherapy outcomes in terms of LC/LF and ARE detection, on independent external data. The results of Kaplan–Meier time‐to‐event analyses demonstrated that the proposed framework could replicate clinical radiotherapy outcome assessments in timely detecting LF and ARE after SRS across various tumor sizes.

Given its strong performance in tumor segmentation, longitudinal monitoring, and automatic outcome assessment, the proposed framework holds significant potential as a clinical decision support tool for precision radiotherapy. Timely evaluation of radiotherapy outcome in BM is clinically essential since tumors with LF and ARE require quite different, yet time‐sensitive, treatments. Automating this process could streamline the clinical workflow in neuro‐oncology, reduce the potential for human error, and improve standardized radiotherapy outcome assessments.

The current standard for radiotherapy outcome assessment in BM is based on changes in tumor longest diameter measured on 3D MRI, following the RANO‐BM criteria. While the RANO‐BM working group has provided guidelines for outcome assessment based on the changes in tumor volume,^9^ its proposed criteria for volumetric analysis are incomplete due to lack of research supporting specific recommendations. This limitation has hindered wide‐spread adoption of volumetric analysis for radiotherapy outcome assessment in clinical trials. The automated framework proposed in this study can facilitate future research in this domain and pave the way toward a volumetric radiotherapy response assessment paradigm.

The proposed automated outcome assessment framework detects the presence of ARE after radiotherapy based on the pattern of tumor size changes on standard serial MRI with acceptable accuracy compared to clinical assessment. However, conventional serial MRI alone may not always be sufficient for definitive diagnosis of ARE versus tumor progression. Additional radiological insights, such as T1/T2 matching^12^ or perfusion MRI,^40^ along with other clinical evidence and histological confirmation may sometimes be necessary to diagnose ARE in the clinic. Considering the performance of the proposed framework in detecting LC/LF and ARE outcomes on serial MRI, it can be applied as an effective decision support system to triage complicated cases that require further assessment by neuro‐oncologists. Advanced imaging techniques including positron emission tomography (PET)^41^ and chemical exchange saturation transfer (CEST) MRI^42^ have shown high diagnostic accuracy in distinguishing ARE from tumor progression after radiotherapy. Future research may focus on integrating complementary imaging modalities, including perfusion and diffusion MRI, CEST, or PET, with the automated radiotherapy outcome assessment framework to improve accuracy in distinguishing ARE from tumor progression.

## CONFLICT OF INTEREST STATEMENT

Hany Soliman, Arjun Sahgal, and Ali Sadeghi‐Naini are inventors of a pending patent application on “System and methods for automatic assessment of radiotherapy outcome in tumors using longitudinal tumor segmentation on serial MRI.”
